# Skeletal Microstructure in Caribbean Hispanic Women

**DOI:** 10.1002/jbm4.10725

**Published:** 2023-03-01

**Authors:** Sanchita Agarwal, Carmen Germosen, Mariana Bucovsky, Ivelisse Colon, Nayoung Kil, Marcella Walker

**Affiliations:** ^1^ Division of Endocrinology Columbia University Irving Medical Center New York NY USA

**Keywords:** ETHNICITY, FAILURE LOAD, FRACTURE, HISPANIC, MICROSTRUCTURE, RACE

## Abstract

Hispanic individuals are underrepresented in skeletal research. Bone mineral density (BMD) and fracture data are conflicting. We investigated skeletal health in elderly Caribbean Hispanic (HW), non‐Hispanic white (NHW), and non‐Hispanic black (NHB) women in a population‐based study in New York City. We utilized high‐resolution peripheral quantitative CT (HRpQCT), dual‐energy X‐ray absorptiometry (DXA), and finite element analysis (FEA). Of 442, 48.4% were HW, 21.3% NHW, and 30.3% NHB. Adjusted analyses are shown. Compared to NHW, HW had 8.5% (*p* < 0.01) lower spine areal BMD (aBMD) and 5.1% lower trabecular bone score (TBS). The frequency of morphometric vertebral fractures did not differ between HW and NHW. By HRpQCT, HW had 2.9% higher cortical (Ct) volumetric BMD (vBMD), 7.9% greater Ct area (Ct.Ar) and 9.4% greater Ct thickness (Ct.Th) at the radius compared to NHW. Results were similar at the tibia but trabecular microstructure tended to be worse. Ultimately, failure load (FL) did not differ between HW and NHW at either site. aBMD was 3.8% to 11.1% lower at the spine, femoral neck, and radius in HW compared to NHB (all *p* < 0.001) and vertebral fractures were twice as common. Compared to NHB, HW had 7.7% to 10.3% lower Ct.Ar at both the radius and tibia as well as 8.4% lower total vBMD, 6.3% lower trabecular number, and 10.3% lower Ct.Th at the tibia associated with 18.2% and 12.5% lower FL at both sites, respectively. In conclusion, HW had lower spine aBMD and TBS versus NHW women, whereas microstructural differences at the radius and tibia were small and not associated with differences in FL. In contrast, HW had lower aBMD, as well as deteriorated radial and tibial microstructure associated with worse FL compared to NHB women. Our results provide insight into racial/ethnic differences in skeletal health, adding to data that may be used to improve osteoporosis screening and treatment in HW. © 2023 The Authors. *JBMR Plus* published by Wiley Periodicals LLC on behalf of American Society for Bone and Mineral Research.

## Graphical abstract

Racial/ethnic differences in bone microstructure.

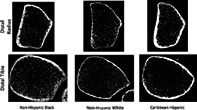

## Introduction

Areal BMD (aBMD) as measured by dual‐energy X‐ray absorptiometry (DXA) varies by race and ethnicity.^(^ ^1^ ^)^ There are almost 60 million Hispanic‐Americans in the United States and this number is growing.^(^ ^2^ ^)^ Hispanic men and women are underrepresented in studies of skeletal health. The available studies have often focused on Mexican Americans.^(^ ^3^, ^4^ ^)^ The US Hispanic population is, however, very diverse.^(^ ^2^ ^)^ Different Hispanic subgroups vary in socioeconomic, demographic, lifestyle, and other factors that influence bone indices.^(^ ^2^ ^)^ The few studies that examined skeletal health in other Hispanic subgroups do suggest important differences by Hispanic origin.^(^ ^5^, ^6^, ^7^ ^)^ Some studies have ignored Hispanic ethnic origin.^(^ ^6^, ^8^ ^)^ As a result, studies vary in their cohort composition, leading to conflicting data regarding aBMD and fracture risk. Some studies indicate higher, equivalent or lower aBMD in Hispanic compared to non‐Hispanic white (NHW) men and women.^(^ ^3^, ^9^, ^10^, ^11^ ^)^ Similarly, risk of incident fracture in Hispanics has been reported to be lower or similar compared to NHW individuals.^(^ ^8^, ^12^, ^13^, ^14^, ^15^ ^)^

A recent work emphasized aggregation into broad ethnic or racial groups (ie, Hispanic or Asian) would likely conceal important differences in bone indices. The article highlighted the need for research within racial/ethnic groups by origin subgroup or background.^(^ ^5^ ^)^ Caribbean Hispanics (individuals of Cuban, Puerto Rican, and Dominican descent) make up the largest subgroup in the United States after Mexican Americans, yet little skeletal data is available in this group.^(^ ^2^ ^)^ Without accurate data regarding skeletal characteristics that influence fracture risk (eg, aBMD and microstructure) in specific Hispanic origin groups, it is impossible to create effective screening and treatment strategies that are relevant to these subgroups and reduce health disparities.^(^ ^5^ ^)^

The aim of this analysis was to investigate skeletal health in Hispanic women of Caribbean origin (HW) compared to non‐Hispanic White (NHW) and non‐Hispanic black (NHB) women in the context of a multiethnic, population‐based study using high‐resolution peripheral quantitative CT (HRpQCT) and other state‐of‐the‐art skeletal imaging methodologies. HRpQCT has provided insight into understanding racial differences in fracture risk, not entirely explained by DXA in both NHB and Asian women compared with NHW women.^(^ ^16^, ^17^, ^18^ ^)^ To our knowledge, only one prior study has assessed skeletal microstructure in HW.^(^ ^19^ ^)^ This single study included only a convenience sample of 33 HW and was too small to draw definitive conclusions with relevance to the broader population.^(^ ^19^ ^)^ We hypothesized that women of Caribbean Hispanic descent would have microstructural values (primary outcome cortical thickness) intermediate to NHW and NHB women due to African admixture.^(^ ^20^ ^)^

## Subjects and Methods

### Design

This cross‐sectional ancillary analysis compared skeletal health data in elderly HW, NHW, and NHB women who were participating in a population‐based longitudinal cohort study of aging. The Columbia University Irving Medical Center (CUIMC) Institutional Review Board approved this study and all participants provided written informed consent.

### Study population

The Washington Heights Hamilton Heights Inwood Community Aging Project (WHICAP) is a National Institutes of Health (NIH)‐funded community‐based prospective cohort study of aging in elderly, urban‐dwelling ambulatory residents (age > 65 years) living in Northern Manhattan. The design and recruitment for the study have been published previously.^(^ ^21^, ^22^ ^)^ Briefly, a probability sample of Medicare recipients, age ≥ 65 years without dementia from three zip codes in Northern Manhattan was recruited beginning in 1992 and enriched with further recruitment subsequently. Returning participants capable of consenting were invited to this ancillary study assessing bone health. Those who agreed to participate underwent a one‐time evaluation with DXA, HRpQCT, and a questionnaire regarding their health and fracture history. All races and ethnicities were invited, but given the geographical location, most participants were Caribbean Hispanic, NHB, and NHW. This cross‐sectional analysis includes women enrolled to the ancillary study who had imaging between January 2019 and May 2022 and self‐identified as NHB, NHW or Hispanic: NHB (*n* = 134), NHW (*n* = 94) and Caribbean Hispanic (*n* = 214). Men and other races/ethnicities and Hispanic subgroups were excluded from this analysis due to the smaller numbers enrolled.

### DXA

aBMD was measured with a Hologic instrument (Hologic, Waltham, Massachusetts, USA). aBMD measurements were obtained at the lumbar spine L_1_–L_4_ (LS), femoral neck (FN), total hip (TH), and 1/3 radius. *T*‐scores were obtained using the manufacturer's white reference norms. Participants were scanned at all three skeletal sites unless hardware precluded the analysis of aBMD. We excluded vertebra with hardware or other artifacts from the analysis of aBMD at the spine. In vivo precision is 1.28% at the LS, 1.36% at the hip, and 0.70% for the distal radius (1/3 site).^(^ ^23^ ^)^

Spine trabecular bone score (TBS) was calculated from subjects' spine DXA image using TBS iNsight software (version 3.0.3.0; Medimaps, Geneva, Switzerland)^(^ ^24^ ^)^ if the spine image was evaluable. Lateral spine scans for vertebral fracture assessment (VFA) were acquired from T_4_ to L_5_ when evaluable. Participants were categorized as having vertebral fractures in the imaged spine using the Genant semiquantitative method: mild, moderate, and severe compression fractures were defined as a 20% to 25%, 26% to 40% or >40% reduction in vertebral height, respectively.^(^ ^25^ ^)^

### HRpQCT

HRpQCT was performed with an XtremeCT II scanner (Scanco Medical, Brüttisellen, Switzerland), which uses a microfocus X‐ray source (68 kVp voltage, 900 μA current, 43 second integration time) scanning a region 10.2 mm long along the axis of the long bone resulting in volume of interest (VOI) of 60.7 μm isotropic voxel size. The non‐dominant distal radius and tibia were scanned unless there was a prior fracture or metal implant. The region of interest was defined on a two‐dimensional (2D) scout view by placing a reference line at the endplate: proximal endplate for radius and distal endplate for tibia. Images were acquired using a relative offset from the reference line; radius scans at 4% of limb length and tibia at 7.3%. We also scanned the tibia at a more proximal diaphyseal region at 30%, composed almost entirely of cortical bone. A single highly trained operator acquired and analyzed all scans. Scans with a motion score >3 (scale 1–5) were excluded from the analysis.^(^ ^26^ ^)^ We used the manufacturer's standard method to filter and binarize the HRpQCT images. An automated segmentation algorithm was used to segment the cortical and trabecular regions.^(^ ^27^ ^)^ We assessed area; density—total, trabecular (Tb), and cortical (Ct) vBMD; microstructure—trabecular number (Tb.N), thickness (Tb.Th), separation (Tb.Sp), standard deviation of the trabecular separation (Tb.1/N.SD), cortical thickness (Ct.Th), and cortical porosity (Ct.Po). In vivo short‐term reproducibility (CV) is between 0% and 5% for all measures except Ct.Po.

### FEA

Whole‐bone FL was estimated from the HRpQCT images using FEA based on a voxel conversion approach. We simulated an axial compression on each radius and tibia model up to 1% strain using a homogeneous Young's modulus of 10 GPa and Poisson's ratio of 0.3. We used micro‐FEA (μFEA) solver provided by the manufacturer (Scanco Medical FE‐software v1.13; Scanco Medical) to solve the models.

### Questionnaire and clinical evaluation

Information regarding past medical history, lifestyle, medications, and fall recall was collected by questionnaire. Daily calcium and vitamin D intake from diet and supplements was assessed with a validated standardized food frequency questionnaire.^(^ ^28^ ^)^ Physical activity was assessed with the physical activity scale for the elderly.^(^ ^29^ ^)^ Weight and height were measured by balance beam and a wall‐mounted, calibrated Harpenden stadiometer, respectively.

### Statistics

Descriptive statistics were expressed as means and SDs or absolute (*n*) and relative (%) frequency. Between‐group differences in continuous variables were evaluated using one‐way analysis of variance (ANOVA) with post‐hoc Tukey's test. Between‐group differences in categorical variables were evaluated using Pearson's chi‐square test with 2×N contingency table comparisons for post‐hoc test between races. Adjusted analyses were conducted using analysis of covariance controlling for age, weight, menopause age, calcium intake, physical activity, smoking, diabetes, current osteoporosis treatment, diabetes, thiazide use, and anti‐depressant use. Covariates were selected on the basis of prior associations, known biological mechanisms, and/or between‐group differences. In additional models, we assessed the influence of socioeconomic factors. We did not adjust for height because between‐group differences in limb length were addressed by utilizing a relative rather than standard offset for HRpQCT and 1/3‐radius DXA measurement. Analyses were performed using Python version 3.7.4 (https://www.python.org/downloads/release/python-374/) and R version 3.6.3 (R Foundation for Statistical Computing, Vienna, Austria; https://www.r-project.org/). A two‐tailed *p*‐value <0.05 was considered statistically significant.

## Results

### Demographics and clinical characteristics

The racial distribution of the cohort was 48.4% HW, 21.3% NHW, and 30.3% NHB. Mean age of the cohort was 77.1 ± 6.0 years and there were no between‐group differences in age. As shown in Table 1, comparing HW to NHW women, height was lower whereas BMI was higher in HW. HW were less likely to be college‐educated with lower household income compared to NHW women (*p* < 0.001). Rates of historical clinical osteoporotic fractures and nonvertebral osteoporotic fractures in HW were approximately one‐half that of NHW women (*p* < 0.001). Specifically, forearm (6.1% versus 12.8%; *p* < 0.05), lower leg (5.6% versus 9.6%), foot (0.9% versus 5.3%; *p* < 0.05), and knee fractures (0.5% versus 4.3%; *p* < 0.05) were less frequent in HW compared to NHW women. The prevalence of family history of hip fracture in HW was less than half that of NHW women (*p* < 0.01). Physical activity score was lower in Hispanic compared to NHW women (*p* < 0.001). Prevalence of diabetes was two to three times higher in Hispanic compared with NHW women (*p* < 0.001). Thiazide use was more common in Hispanic than NHW women (*p* < 0.01).

**Table 1:** Racial/Ethnic Differences in Demographic and Clinical Characteristics

Characteristic	NHB (*n* = 134)	NHW (*n* = 94)	HW (*n* = 214)	*p*
Age (years)	77.0 ± 6.2	77.4 ± 4.8	77.1 ± 6.3	0.82
Ethnicity (% Hispanic)	0	0	100	**<0.001** ^**b**^ ^,^ ^**c**^
Weight (kg)	79.9 ± 18.7	67.5 ± 15.1	69.8 ± 14.2	**<0.001** ^**a**^ ^,^ ^**b**^
Height (m)	1.61 ± 0.06	1.58 ± 0.07	1.54 ± 0.08	**<0.001** ^**b**^ ^,^ ^**c**^
BMI (kg/m^2^)	30.9 ± 6.9	26.9 ± 5.7	29.4 ± 5.8	**<0.001** ^**a**^ ^,^ ^**c**^
Place of birth (%)				**<0.001** ^**a**^ ^,^ ^**b**^ ^,^ ^**c**^
United States	95.5	84.0	3.3	
Outside United States	4.5	16.0	96.7	
Highest education level (%)				**<0.001** ^**a**^ ^,^ ^**b**^ ^,^ ^**c**^
Grammar school	10.5	0.0	62.6	
High school	52.2	11.7	27.6	
College or advanced professional degree	37.3	88.3	9.8	
Household income (%)				**<0.001** ^**a**^ ^,^ ^**b**^ ^,^ ^**c**^
<$50k	86.2	31.8	98.1	
$50k to <$200k	13.8	63.6	1.9	
>$200k	0.0	4.6	0.0	
Calcium intake (mg/d)	1063 ± 593	1391 ± 610	1222 ± 565	**<0.001** ^**a**^ ^,^ ^**b**^
Vitamin D intake (IU/d)	1578 ± 1855	1712 ± 1571	1835 ± 2172	0.49
Prior clinical fractures (%)	45	65	40	**<0.001** ^**a**^ ^,^ ^**c**^
Prior clinical fractures (*n*)	1.4 ± 1.1	2.2 ± 1.41	1.5 ± 0.81	**<0.001** ^**a**^ ^,^ ^**c**^
Clinical osteoporotic fractures (%)	19	42	22	**<0.001** ^**a**^ ^,^ ^**c**^
Clinical nonvertebral osteoporotic fractures (%)	19	40	20	**<0.001** ^**a**^ ^,^ ^**c**^
Menopause age (years)	47.7 ± 7.0	50.0 ± 4.7	47.8 ± 7.2	**0.02** ^**a**^ ^,^ ^**c**^
Percent with fall in past 12 months (%)	31	35	25	0.17
Falls in past 12 months (*n*)	1.8 ± 1.9	2.8 ± 4.0	1.9 ± 2.3	0.25
Family history of hip fracture (%)	6	19	7	**<0.01** ^**a**^ ^,^ ^**c**^
Physical activity score	78.9 ± 40.9	95.8 ± 41.7	73.8 ± 48.0	**<0.001** ^**a**^ ^,^ ^**c**^
Current Smokers (%)	7	2	2	**0.04** ^**b**^
Pack‐years	9.4 ± 16.4	8.3 ± 18.2	5.0 ± 14.0	**0.03** ^**b**^
Diabetes (%)	29	11	36	**<0.001** ^**a**^ ^,^ ^**c**^
Current osteoporosis treatment (%)	3	11	15	**<0.01** ^**a**^ ^,^ ^**b**^
Past osteoporosis treatment (%)	12	26	25	**<0.01** ^**a**^ ^,^ ^**b**^
Thiazide (%)	31	12	31	**<0.01** ^**a**^ ^,^ ^**c**^
Anti‐depressants (%)	7	20	14	**0.01** ^**a**^

*Note*: Data are shown as mean ± SD or %. Values of *p* < 0.05 were considered significant and are shown in bold.

Abbreviations: HW = Hispanic; NHB = non‐Hispanic black; NHW = non‐Hispanic white.

^a^ *p* < 0.05 black versus white.

^b^ *p* < 0.05 black versus Hispanic.

^c^ *p* < 0.05 white versus Hispanic.

Comparing HW to NHB women, weight and height were lower in HW (both *p* < 0.001). HW had lower education and household income compared to NHB women (*p* < 0.001). Calcium intake was higher in HW compared to NHB women (*p* < 0.05). Smoking was less frequent in HW compared to NHB women, whereas osteoporosis treatment was more common. There were no differences in historical fractures, family history, physical activity, diabetes, or other medication use. Differences between NHW and NHB women are also shown in Table 1.

### aBMD by DXA

**Fig. 1:**
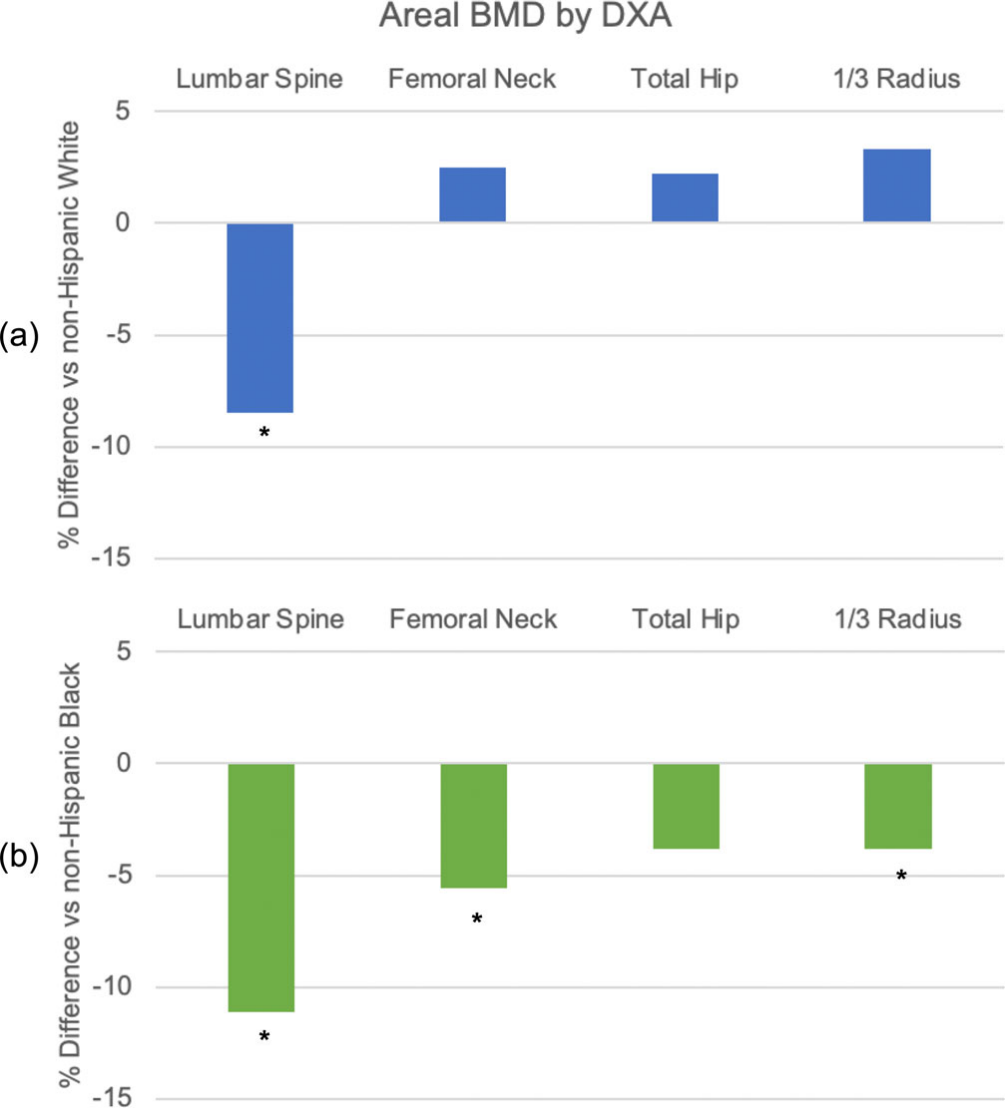
Adjusted racial/ethnic differences in aBMD in Caribbean Hispanic compared to (a) NHW (blue) and (b) NHB (green) women. *p < 0.05.

As shown in Table 2, comparing HW to NHW women, unadjusted aBMD was 5.4% lower at the LS, but 4.9% higher at the 1/3 radius. After adjusting for covariates, HW had 8.5% lower aBMD at the LS, but no differences at other sites compared to NHW women (Fig. 1).

**Table 2:** Racial/Ethnic Differences in aBMD, Body Composition, VFA, and TBS by DXA

Parameter	NHB (*n* = 134)	NHW (*n* = 94)	HW (*n* = 214)	*p*	Adjusted *p* ^#^
LS *T*‐score	0.2 ± 1.9	−0.6 ± 1.5	−1.1 ± 1.5	**<0.001** ^**a**^ ^,^ ^**b**^ ^,^ ^**c**^	**<0.001** ^**b**^ ^,^ ^**c**^
FN *T*‐score	−1.0 ± 1.2	−1.8 ± 0.9	−1.6 ± 0.9	**<0.001** ^**a**^ ^,^ ^**b**^	**<0.001** ^**a**^ ^,^ ^**b**^
TH *T*‐score	−0.8 ± 1.2	−1.5 ± 1.0	−1.3 ± 1.0	**<0.001** ^**a**^ ^,^ ^**b**^	**0.02** ^**a**^
1/3‐Radius *T*‐score	−0.6 ± 1.5	−1.9 ± 1.3	−1.3 ± 1.4	**<0.001** ^**a**^ ^,^ ^**b**^ ^,^ ^**c**^	**<0.001** ^**a**^ ^,^ ^**b**^
Percent with ≥1 spine fracture(s) detected by VFA (%)	6 (*n* = 7)	13 (*n* = 12)	13 (*n* = 26)	0.17	**<0.01**
Number of spine fractures in those with ≥1 spine fracture(s) (*n*)	1.1 ± 0.4	1.3 ± 0.7	1.4 ± 0.8	0.78	0.24
TBS	1.213 ± 0.132	1.260 ± 0.116	1.193 ± 0.118	**<0.001** ^**a**^ ^,^ ^**c**^	**<0.01** ^**c**^

*Note*: Data are shown as mean ± SD or %. Values of *p* < 0.05 were considered significant and are shown in bold.

Abbreviations: HW = Hispanic; NHB = non‐Hispanic black; NHW = non‐Hispanic white.

^a^ *p* < 0.05 black versus white.

^b^ *p* < 0.05 black versus Hispanic.

^c^ *p* < 0.05 white versus Hispanic.

^#^ Model adjusted for age, weight, calcium intake, current osteoporosis treatment, physical activity score, menopause age, smoking, diabetes, thiazide, and anti‐depressant use.

Compared to NHB women, HW had 7.6% to 15.2% lower aBMD at all sites prior to adjustment for covariates. After adjustment (Table 2, Fig. 1), HW remained 3.8% to 11.1% lower at all sites except the TH. Comparing, NHW and NHB women, NHW women had lower aBMD at all sites prior to adjustment. Differences remained significant after adjustment for covariates except at the LS.

### VFA and TBS by DXA

Comparing HW and NHW women, prevalence of vertebral fracture by VFA did not differ before or after adjustment for covariates (Table 2). In HW, unadjusted and adjusted TBS was 5.6% and 5.1% lower, respectively, compared to NHW women.

Comparing to NHB women, rates of compression fracture in HW were double that of NHB women after adjustment. TBS in HW did not differ from NHB women before or after adjustment. Comparing NHW and NHB women, TBS was higher in NHW women before, but not after adjustment (Table 2).

### HRpQCT

#### Radius

**Fig. 2:**
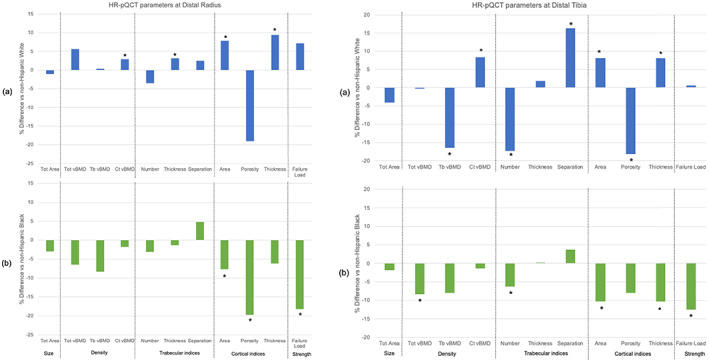
Adjusted racial/ethnic differences in HRpQCT and FEA indices at the (*A*) radius and (*B*) tibia in Caribbean Hispanic compared to (a) NHW (blue) and (b) NHB (green) women. *p < 0.05.

Differences in skeletal microstructure at the radius and tibia by HRpQCT are shown in Table 3 and Fig. 2. Comparing HW to NHW women, HW had 4.3% thicker trabeculae, 9.7% greater Ct.Ar, as well as 10.4% thicker and 3.6% denser cortices than NHW women before adjustment for covariates. As shown in Fig. 2*A*, after adjustment, differences remained significant (all *p* < 0.01). Ct.Po was lower in Hispanic versus NHW women before adjustment only. Ultimately, FL did not differ between HW and NHW women before or after adjustment.

**Table 3:** Racial/Ethnic Differences in vBMD, Microstructure and Estimated Mechanical Competence by HRpQCT

Parameter	NHB (*n* = 134)	NHW (*n* = 94)	HW (*n* = 214)	*p*	Adjusted *p* ^#^
Distal radius (4%)					
Tt.Ar (mm^2^)	292 ± 44	278 ± 47	279 ± 41	**0.02** ^**b**^	0.25
Tot.vBMD (mg HA/cm^3^)	255 ± 65	215 ± 55	230 ± 58	**<0.001** ^**a**^ ^,^ ^**b**^	**<0.01** ^**a**^
Tb.vBMD (mg HA/cm^3^)	139 ± 43	121 ± 37	122 ± 38	**<0.001** ^**a**^ ^,^ ^**b**^	0.06
Tb.N (1/mm)	1.30 ± 0.24	1.24 ± 0.25	1.21 ± 0.25	**<0.01** ^**b**^	0.28
Tb.Th (mm)	0.23 ± 0.02	0.22 ± 0.01	0.23 ± 0.02	**<0.01** ^**a**^ ^,^ ^**c**^	**<0.01** ^**a**^ ^,^ ^**c**^
Tb.Sp (mm)	0.76 ± 0.18	0.82 ± 0.22	0.84 ± 0.28	**<0.01** ^**b**^	0.29
Tb.1/N.SD (mm)	0.32 ± 0.13	0.39 ± 0.2	0.38 ± 0.27	**0.01** ^**a**^ ^,^ ^**b**^	0.19
Ct.Ar (mm^2^)	51 ± 11	41 ± 8	46 ± 8	**<0.001** ^**a**^ ^,^ ^**b**^ ^,^ ^**c**^	**<0.001** ^**a**^ ^,^ ^**b**^ ^,^ ^**c**^
Ct.vBMD (mg HA/cm^3^)	813 ± 67	766 ± 81	794 ± 76	**<0.001** ^**a**^ ^,^ ^**c**^	**<0.01** ^**a**^ ^,^ ^**c**^
Ct.Po (%)	1.31 ± 0.79	1.25 ± 0.82	1.01 ± 0.68	**<0.001** ^**b**^ ^,^ ^**c**^	**0.03** ^**b**^
Ct.Th (mm)	0.85 ± 0.20	0.69 ± 0.16	0.77 ± 0.18	**<0.001** ^**a**^ ^,^ ^**b**^ ^,^ ^**c**^	**<0.001** ^**a**^ ^,^ ^**c**^
FL (N)	2897 ± 823	2108 ± 620	2320 ± 822	**<0.001** ^**a**^ ^,^ ^**b**^	**<0.001** ^**a**^ ^,^ ^**b**^
Distal tibia (7.3%)					
Tt.Ar (mm^2^)	721 ± 102	713 ± 102	692 ± 91	**0.02** ^**b**^	0.07
Tot.vBMD (mg HA/cm^3^)	253 ± 59	223 ± 46	225 ± 54	**<0.001** ^**a**^ ^,^ ^**b**^	**<0.01** ^**b**^
Tb.vBMD (mg HA/cm^3^)	151 ± 43	153 ± 34	134 ± 39	**<0.001** ^**b**^ ^,^ ^**c**^	**<0.001** ^**c**^
Tb.N (1/mm)	1.24 ± 0.25	1.30 ± 0.20	1.12 ± 0.25	**<0.001** ^**b**^ ^,^ ^**c**^	**<0.001** ^**a**^ ^,^ ^**b**^ ^,^ ^**c**^
Tb.Th (mm)	0.25 ± 0.02	0.25 ± 0.02	0.25 ± 0.02	0.06	0.23
Tb.Sp (mm)	0.82 ± 0.29	0.76 ± 0.16	0.92 ± 0.31	**<0.001** ^**b**^ ^,^ ^**c**^	**<0.001** ^**a**^ ^,^ ^**c**^
Tb.1/N.SD (mm)	0.37 ± 0.31	0.34 ± 0.19	0.44 ± 0.41	**0.03** ^**c**^	0.09
Ct.Ar (mm^2^)	113 ± 27	87 ± 20	97 ± 23	**<0.001** ^**a**^ ^,^ ^**b**^ ^,^ ^**c**^	**<0.001** ^**a**^ ^,^ ^**b**^ ^,^ ^**c**^
Ct.vBMD (mg HA/cm^3^)	798 ± 77	724 ± 83	783 ± 82	**<0.001** ^**a**^ ^,^ ^**c**^	**<0.001** ^**a**^ ^,^ ^**c**^
Ct.Po (%)	4.24 ± 1.77	4.57 ± 1.70	3.90 ± 1.68	**<0.01** ^**c**^	**<0.01** ^**c**^
Ct.Th (mm)	1.32 ± 0.33	1.03 ± 0.25	1.15 ± 0.28	**<0.001** ^**a**^ ^,^ ^**b**^ ^,^ ^**c**^	**<0.001** ^**a**^ ^,^ ^**b**^ ^,^ ^**c**^
FL (N)	8787 ± 1930	7203 ± 1575	7383 ± 2437	**<0.001** ^**a**^ ^,^ ^**b**^	**<0.001** ^**a**^ ^,^ ^**b**^
Proximal tibia (30%)					
Ct.Ar (mm^2^)	223 ± 43	202 ± 29	198 ± 36	**<0.001** ^**a**^ ^,^ ^**b**^	**<0.01** ^**b**^
Ct.vBMD (mg HA/cm^3^)	1008 ± 45	985 ± 41	1009 ± 42	**<0.001** ^**a**^ ^,^ ^**c**^	**<0.01** ^**a**^ ^,^ ^**c**^
Ct.Po (%)	1.29 ± 1.0	2.10 ± 1.38	1.38 ± 1.22	**<0.001** ^**a**^ ^,^ ^**c**^	**<0.01** ^**a**^ ^,^ ^**c**^
Ct.Th (mm)	4.42 ± 0.95	4.26 ± 0.78	4.10 ± 0.81	**<0.01** ^**b**^	0.18
FL (N)	13456 ± 2383	11865 ± 1806	11791 ± 2079	**<0.001** ^**a**^ ^,^ ^**b**^	**<0.001** ^**a**^ ^,^ ^**b**^

*Note*: Data are shown as mean ± SD. Values of *p* < 0.05 were considered significant and are shown in bold.

Abbreviations: HA = hydroxyapatite; HW = Hispanic; NHB = non‐Hispanic black; NHW = non‐Hispanic white; Tot.vBMD = total vBMD; Tt.Ar = total area.

^a^ *p* < 0.05 black versus white.

^b^ *p* < 0.05 black versus Hispanic.

^c^ *p* < 0.05 white versus Hispanic.

^#^ Model adjusted for age, weight, calcium intake, current osteoporosis treatment, physical activity score, menopause age, smoking, diabetes, thiazide, and anti‐depressant use.

**Fig. 3:**
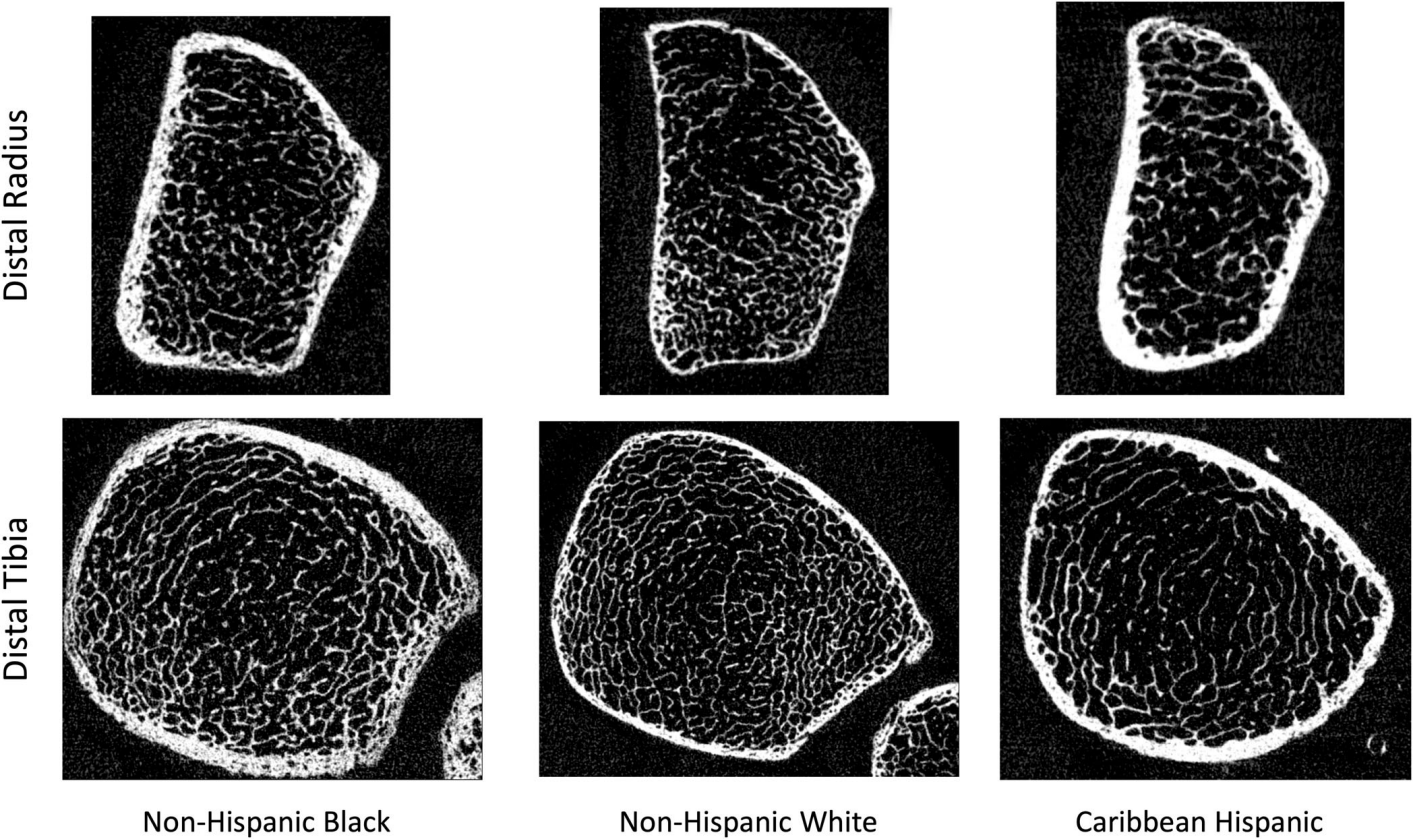
Representative HRpQCT images of the radius (top) and tibia (bottom) in NHB (left), NHW (middle) and HW (right).

Comparing HW to NHB women, HW had 4.6% smaller bone size, 10.8% lower vBMD and less favorable microstructure for all parameters, except Ct.vBMD and Tb.Th, leading to 24.9% lower FL prior to adjustment (Table 3). After adjusting for covariates (Fig. 2*A*), most differences between HW and NHB women were attenuated except for 7.7% lower Ct.Ar and 19.7% lower Ct.Po in HW. Adjusted FL was 18.2% lower in HW versus NHB women. Representative images are shown in Fig. 3.

Comparing NHB to NHW women, NHB women had higher vBMD and more favorable microstructure across all parameters except Tb.N, Tb.1/N.SD, and Ct.Po before and after adjustment. Ultimately, this led to 37.4% higher FL before and 27.4% higher FL after adjustment in NHB women.

#### Tibia

Some similar patterns emerged at the distal tibia (Table 3, Fig. 2*B*). Comparing HW to NHW women, HW had 7.5% higher Ct.vBMD, 10.4% higher Ct.Ar, 10.4% greater Ct.Th, and 17.2% lower Ct.Po before adjustment. As shown in Fig. 2*B*, these differences remained significant after adjustment. In contrast, unadjusted Tb.vBMD was 14.2% lower; Tb.N was 16.1% lower, and Tb.Sp was 17.4% higher in Hispanic compared to NHW women. These differences remained significant after adjustment (Fig. 2*B*). Ultimately, cortical advantages were offset by trabecular disadvantages and FL did not differ before or after adjustment for covariates between Hispanic and NHW. At the 30% diaphyseal site, HW had 2.4% higher Ct.vBMD and 52.2% lower Ct.Po compared to NHW women before adjusting for covariates. These differences remained significant after adjustment, but were not associated with greater FL.

Comparing HW to NHB women, HW had 4.3% smaller bone size; 12.3% lower total vBMD, 12.3% lower Tb.vBMD, 10.7% lower Tb.N; 10.9% greater Tb.Sp; 15.9% greater Tb.1/N.SD and 16.2% lower Ct.Ar and 14.8% Ct.Th, which were associated with 19.0% lower FL before adjustment for covariates. After adjusting for covariates, differences in total vBMD, Tb.N, Ct.Ar, and Ct.Th persisted, and were associated with 12.5% lower FL in HW (Fig. 2*B*). Similarly, at the 30% diaphyseal site, HW had 12.8% lower Ct.Ar and 14.1% lower FL before adjustment compared to NHB women. Differences persisted after adjustment.

Comparing NHB and NHW women, trabecular indices did not differ before adjustment. After adjustment, NHB women had 9.4% lower Tb.N and 15.0% higher Tb.Sp compared to NHW women. Before adjustment, NHW women had 29.6% lower Ct.Ar, 10.3% lower Ct.vBMD, and 28.2% lower Ct.Th. Differences remained significant after adjustment. These differences were associated with 22.0% and 13.2% higher FL before and after adjustment in NHB women, respectively.

#### Comparison of Caribbean Hispanic subgroups

We further categorized the Caribbean Hispanic women (*n* = 214) into subgroups based on their origin: Dominican (*n* = 173), Puerto Rican (*n* = 22), and Cuban (*n* = 19). In this subgroup analysis, Dominican women had lower weight compared to Puerto Rican women but did not differ in age or height. After adjusting for weight, Dominican women had 19.2% lower Tb.vBMD compared to Cuban women and 14.6% lower Tb.N compared to Puerto Rican women at the radius (all *p* < 0.05). At the tibia, Dominican women had 22.1% lower Tb.vBMD and 19.6% greater Tb.Sp compared to Puerto Rican women and lower Tb.N compared to both Puerto Rican and Cuban women.

#### Analysis of socioeconomic factors

Because education and household income differed between race/ethnicities, they were added as additional covariates to separate models to explore their influence. Differences in TBS and Ct.Po at both radius and tibia, and Tb.vBMD and microstructure at the tibia were attenuated and no longer significant. On the other hand, Tb.vBMD at the radius became significantly lower in NHW compared to NHB women (*p* < 0.01) (data not shown). Other differences persisted.

## Discussion

To our knowledge, this is the first population‐based and largest study investigating skeletal microstructure and mechanical competence in HW using HRpQCT. We showed that HW tended to have advantageous cortical microstructure by HRpQCT compared to NHW women but worse trabecular indices at some sites. Differences did not result in lower mechanical competence by FEA. In contrast, both trabecular and cortical indices along with FL were lower in HW compared to NHB women. Additionally, HW had lower LS aBMD and TBS compared to NHW women and lower aBMD at all sites compared to NHB women. Skeletal microstructure (measured by HRpQCT or TBS) and FEA have been shown to predict fracture independently of aBMD in other studies.^(^ ^30^, ^31^ ^)^ Our findings help clarify data regarding skeletal characteristics that may influence fracture in this particular Hispanic subgroup.

Racial differences between HW and NHW women varied to some extent by skeletal site. Although cortical indices tended to be slightly more advantageous in HW at the radius and tibia compared to NHW, trabecular indices were lower at the tibia only in HW. The trabecular disadvantages were offset by more mechanically favorable cortical indices, leading to similar FL at both sites. The racial differences with regard to the trabecular versus cortical compartment detected by HRpQCT are consistent with the site‐specific differences detectable by DXA. Compared to NHW women, aBMD and TBS were lower at the trabecular rich LS in HW, but aBMD was similar at the hip and higher at the 1/3‐radius, which have more cortical bone.

These findings may have implications for osteoporosis screening and treatment. Though recent data suggest improvement, HW have historically been less likely to receive DXA screening than NHW women.^(^ ^5^, ^32^, ^33^ ^)^ This may be due to misconceptions among patients or providers.^(^ ^5^, ^34^, ^35^ ^)^ Additionally, the Fracture Risk Assessment Tool (FRAX) tool computes a lower risk of fracture for an individual with the same absolute aBMD designated as “Hispanic” than a NHW individual. Though little data is available regarding disparities in osteoporosis treatment in Hispanic populations, FRAX calculations adjusted for “Hispanic” ethnicity might deter treatment among Hispanic patients. This practice runs counter to expectations regarding fracture based on our aBMD, TBS, and HRpQCT results. Thus, our findings add to the literature facilitating public and provider knowledge regarding skeletal health in the Caribbean Hispanic population and ultimately may help improve screening and treatment.

We also sought to assess the influence of socioeconomic factors. Some racial/ethnic differences in skeletal microstructure (particularly trabecular deterioration) were attenuated and no longer significant when socioeconomic factors were included in the models. This suggests that some racial/ethnic differences in skeletal health are not related to the genetic background, but acquired (economic status, nutritional status, etc.). We cannot ascertain the specific socioeconomic differences responsible using the current data and this requires further study.

We also aimed to compare bone parameters and FL within the Caribbean Hispanic group by stratifying them based on country of origin. We found that Dominican women tended to have worse trabecular parameters at both sites compared to the other groups, whereas there was no difference in cortical parameters. This finding requires further study given the limited number of Puerto Rican and Cuban individuals.

Our results differ in some ways from a prior smaller study at our own institution investigating skeletal microstructure in Caribbean Hispanic women, Zhou and colleagues^(^ ^19^ ^)^ found HW had worse trabecular microstructure at the tibia compared to white women, but no cortical advantages, ultimately leading to lower stiffness. Our studies have several notable differences that may explain incongruencies. The prior study was a small convenience‐based sample, not a population‐based sample; used a standard offset to acquire HRpQCT images rather than relative offset, the latter of which can account for height/limb length differences; used a first, rather than second generation HRpQCT.^(^ ^36^ ^)^; and did not control for racial/ethnic differences in comorbidities and medications that might influence skeletal health, other than body mass index (BMI). Our HRpQCT findings with regard to differences between NHB and NHW women are similar to those described by Putman and colleagues.^(^ ^37^ ^)^

Our results comparing aBMD in NHW and Caribbean Hispanic are consistent with prior studies showing lower LS aBMD in Mexican Americans compared to NHW women.^(^ ^5^, ^38^, ^39^, ^40^ ^)^ aBMD differences between Caribbean Hispanic and NHB women are generally congruent with prior studies indicating lower aBMD or a higher prevalence of osteoporosis in Mexican American women than black women.^(^ ^5^, ^38^, ^39^, ^40^, ^41^ ^)^ Similar to Looker and colleagues,^(^ ^42^ ^)^ we found lower TBS in HW compared to NHW women, although they studied a different Hispanic subgroup, Mexican Americans.

Despite lower aBMD and TBS in HW versus NHW women in our study, we found prevalent morphometric vertebral fracture rates to be comparable in these groups. This result should be interpreted with caution, due to the cross‐sectional nature of the study and small number of vertebral fractures. Although results are similar to a smaller prior cross‐sectional study and provide information in a larger population‐based sample, prospective studies are needed to make definitive conclusions regarding racial/ethnic differences in vertebral fracture risk between NHW and HW.^(^ ^43^ ^)^

Our study has some limitations. Although this analysis represents the largest study assessing skeletal microstructure in HW and is comparable or larger than prior HRpQCT studies in NHB and NHW women, it remains relatively small in comparison to population‐based studies of aBMD. We had 80% power with a two‐tailed alpha of 5% to detect a 0.34‐SD difference in cortical thickness and FL with the current sample size. It is possible we failed to detect differences of a smaller magnitude. We are also unable to assess incident fracture risk. Our study also has several strengths. Participants were enrolled from a population‐based cohort, which limits selection bias and increases the generalizability of these findings. We had comprehensive information on skeletal covariates and thoroughly assessed musculoskeletal health with multiple modalities. We also assessed HRpQCT using a second‐generation instrument and relative offset to ensure the same region of interest was assessed across the cohort to account for differences in height and limb length. Additionally, we also scanned participants at a proximal tibial site, providing further insight into the effects on cortical bone.

In conclusion, HW had lower spine aBMD and TBS versus NHW women, whereas microstructural differences at the radius and tibia were small and not associated with differences in FL. In contrast, HW had lower aBMD, as well as deteriorated radial and tibial microstructure associated with worse FL compared to NHB women. Our results provide insight into underlying racial/ethnic differences in skeletal health and add to data that may improve osteoporosis screening and treatment in Caribbean Hispanic women.

## Author Contributions

**Sanchita Agarwal:** Data curation; formal analysis; methodology; writing – original draft; writing – review and editing. **Carmen Germosen:** Data curation; writing – review and editing. **Mariana Bucovsky:** Data curation; project administration; supervision; writing – review and editing. **Ivelisse Colon:** Data curation; project administration; writing – review and editing. **Nayoung Kil:** Data curation; writing – review and editing. **Marcella D Walker:** Conceptualization; data curation; funding acquisition; project administration; supervision; writing – review and editing.

## Conflicts of Interest

The authors declare no conflicts of interest.

### Peer Review

The peer review history for this article is available at https://publons.com/publon/10.1002/jbm4.10725.

## References

[1] Cauley JA , Fullman RL , Stone KL , et al. Factors associated with the lumbar spine and proximal femur bone mineral density in older men. Osteoporos Int. 2005;16(12):1525–1537. 15889316 10.1007/s00198-005-1866-8

[2] Noe‐Bustamante L . Key facts about U.S. Hispanics and their diverse heritage. Pew Research Center; 2019. Available from: https://www.pewresearch.org/fact-tank/2019/09/16/key-facts-about-u-s-hispanics/

[3] Looker AC , Melton LJ 3rd , Borrud LG , Shepherd JA . Lumbar spine bone mineral density in US adults: demographic patterns and relationship with femur neck skeletal status. Osteoporos Int. 2012;23(4):1351–1360. 21720893 10.1007/s00198-011-1693-z

[4] Looker AC . Femur neck bone mineral density and fracture risk by age, sex, and race or Hispanic origin in older US adults from NHANES III. Arch Osteoporos. 2013;8:141. 23715737 10.1007/s11657-013-0141-4

[5] Noel SE , Santos MP , Wright NC . Racial and ethnic disparities in bone health and outcomes in the United States. J Bone Miner Res. 2021;36(10):1881–1905. 34338355 10.1002/jbmr.4417 PMC8607440

[6] Araujo AB , Travison TG , Harris SS , Holick MF , Turner AK , McKinlay JB . Race/ethnic differences in bone mineral density in men. Osteoporos Int. 2007;18(7):943–953. 17340219 10.1007/s00198-006-0321-9

[7] Noel SE , Mangano KM , Griffith JL , Wright NC , Dawson‐Hughes B , Tucker KL . Prevalence of osteoporosis and low bone mass among Puerto Rican older adults. J Bone Miner Res. 2018;33(3):396–403. 29044768 10.1002/jbmr.3315 PMC5840013

[8] Barrett‐Connor E , Siris ES , Wehren LE , et al. Osteoporosis and fracture risk in women of different ethnic groups. J Bone Miner Res. 2005;20(2):185–194. 15647811 10.1359/JBMR.041007

[9] Taaffe DR , Villa ML , Holloway L , Marcus R . Bone mineral density in older non‐Hispanic Caucasian and Mexican‐American women: relationship to lean and fat mass. Ann Hum Biol. 2000;27(4):331–344. 10942342 10.1080/03014460050044829

[10] Looker AC , Melton LJ 3rd , Harris TB , Borrud LG , Shepherd JA . Prevalence and trends in low femur bone density among older US adults: NHANES 2005‐2006 compared with NHANES III. J Bone Miner Res. 2010;25(1):64–71. 19580459 10.1359/jbmr.090706 PMC3312738

[11] Morton DJ , Barrett‐Connor E , Kritz‐Silverstein D , Wingard DL , Schneider DL . Bone mineral density in postmenopausal Caucasian, Filipina, and Hispanic women. Int J Epidemiol. 2003;32(1):150–156. 12690028 10.1093/ije/dyg024

[12] Liu LH , Chandra M , Gonzalez JR , Lo JC . Racial and ethnic differences in hip fracture outcomes in men. Am J Manag Care. 2017;23(9):560–564. 29087156

[13] Fang J , Freeman R , Jeganathan R , Alderman MH . Variations in hip fracture hospitalization rates among different race/ethnicity groups in new York City. Ethn Dis. 2004;14(2):280–284. 15132215

[14] Bulathsinhala L , Hughes JM , McKinnon CJ , et al. Risk of stress fracture varies by race/ethnic origin in a cohort study of 1.3 million US Army Soldiers. J Bone Miner Res. 2017;32(7):1546–1553. 28300324 10.1002/jbmr.3131

[15] Chang PY , Saechao FS , Lee J , Haskell SG , Frayne SM , Lee JS . Prevalence and risk of fracture diagnoses in women across the adult life span: a national cross‐sectional study. Osteoporos Int. 2016;27(11):3177–3186. 27349559 10.1007/s00198-016-3655-y

[16] Liu XS , Walker MD , McMahon DJ , et al. Better skeletal microstructure confers greater mechanical advantages in Chinese‐American women versus white women. J Bone Miner Res. 2011;26(8):1783–1792. 21351150 10.1002/jbmr.378 PMC3551974

[17] Walker MD , Liu XS , Stein E , et al. Differences in bone microarchitecture between postmenopausal Chinese‐American and white women. J Bone Miner Res. 2011;26(7):1392–1398. 21305606 10.1002/jbmr.352 PMC3558983

[18] Walker M , Shi S , Russo J , et al. A trabecular plate‐like phenotype is overrepresented in Chinese‐American versus Caucasian women. Osteoporos Int. 2014;25(12):2787–2795. 25069706 10.1007/s00198-014-2816-0

[19] Zhou B , Wang J , Stein EM , et al. Bone density, microarchitecture and stiffness in Caucasian and Caribbean Hispanic postmenopausal American women. Bone Res. 2014;2:14016. 26273525 10.1038/boneres.2014.16 PMC4472134

[20] Peralta CA , Li Y , Wassel C , et al. Differences in albuminuria between Hispanics and whites: an evaluation by genetic ancestry and country of origin: the multi‐ethnic study of atherosclerosis. Circ Cardiovasc Genet. 2010;3(3):240–247. 20445135 10.1161/CIRCGENETICS.109.914499 PMC2948758

[21] Tang MX , Cross P , Andrews H , et al. Incidence of AD in African‐Americans, Caribbean Hispanics, and Caucasians in northern Manhattan. Neurology. 2001;56(1):49–56. 11148235 10.1212/wnl.56.1.49

[22] Agarwal S , Germosen C , Kil N , et al. Current anti‐depressant use is associated with cortical bone deficits and reduced physical function in elderly women. Bone. 2020;140:115552. 32730935 10.1016/j.bone.2020.115552 PMC7502521

[23] Bonnick SL , Johnston CC Jr , Kleerekoper M , et al. Importance of precision in bone density measurements. J Clin Densitom. 2001;4(2):105–110. 11477303 10.1385/jcd:4:2:105

[24] Hans D , Barthe N , Boutroy S , Pothuaud L , Winzenrieth R , Krieg MA . Correlations between trabecular bone score, measured using anteroposterior dual‐energy X‐ray absorptiometry acquisition, and 3‐dimensional parameters of bone microarchitecture: an experimental study on human cadaver vertebrae. J Clin Densitom. 2011;14(3):302–312. 21724435 10.1016/j.jocd.2011.05.005

[25] Genant HK , Wu CY , van Kuijk C , Nevitt MC . Vertebral fracture assessment using a semiquantitative technique. J Bone Miner Res. 1993;8(9):1137–1148. 8237484 10.1002/jbmr.5650080915

[26] Sode M , Burghardt AJ , Pialat J‐B , Link TM , Majumdar S . Quantitative characterization of subject motion in HR‐pQCT images of the distal radius and tibia. Bone. 2011;48(6):1291–1297. 21421091 10.1016/j.bone.2011.03.755 PMC3108045

[27] Buie HR , Campbell GM , Klinck RJ , MacNeil JA , Boyd SK . Automatic segmentation of cortical and trabecular compartments based on a dual threshold technique for in vivo micro‐CT bone analysis. Bone. 2007;41(4):505–515. 17693147 10.1016/j.bone.2007.07.007

[28] Walker MD , McMahon DJ , Udesky J , Liu G , Bilezikian JP . Application of high‐resolution skeletal imaging to measurements of volumetric BMD and skeletal microarchitecture in Chinese‐American and white women: explanation of a paradox. J Bone Miner Res. 2009;24(12):1953–1959. 20001598 10.1359/JBMR.090528 PMC2791512

[29] Washburn RA , Smith KW , Jette AM , Janney CA . The physical activity scale for the elderly (PASE): development and evaluation. J Clin Epidemiol. 1993;46(2):153–162. 8437031 10.1016/0895-4356(93)90053-4

[30] Chapurlat R , Bui M , Sornay‐Rendu E , et al. Deterioration of cortical and trabecular microstructure identifies women with osteopenia or normal bone mineral density at imminent and long‐term risk for fragility fracture: a prospective study. J Bone Miner Res. 2020;35(5):833–844. 31821619 10.1002/jbmr.3924 PMC9328422

[31] Fink HA , Langsetmo L , Vo TN , et al. Association of high‐resolution peripheral quantitative computed tomography (HR‐pQCT) bone microarchitectural parameters with previous clinical fracture in older men: the Osteoporotic Fractures in Men (MrOS) study. Bone. 2018;113:49–56. 29751130 10.1016/j.bone.2018.05.005 PMC6040812

[32] Gillespie CW , Morin PE . Trends and disparities in osteoporosis screening among women in the United States, 2008‐2014. Am J Med. 2017;130(3):306–316. 27884649 10.1016/j.amjmed.2016.10.018

[33] Neuner JM , Binkley N , Sparapani RA , Laud PW , Nattinger AB . Bone density testing in older women and its association with patient age. J Am Geriatr Soc. 2006;54(3):485–489. 16551317 10.1111/j.1532-5415.2005.00622.x

[34] Noel SE , Arevalo SP , Mena NZ , et al. Knowledge, attitudes, beliefs, and health behaviors of bone health among Caribbean Hispanic/Latino adults. Arch Osteoporos. 2019;14(1):14. 30719597 10.1007/s11657-019-0566-5 PMC6448586

[35] Hans D , Goertzen AL , Krieg MA , Leslie WD . Bone microarchitecture assessed by TBS predicts osteoporotic fractures independent of bone density: the Manitoba study. J Bone Miner Res. 2011;26(11):2762–2769. 21887701 10.1002/jbmr.499

[36] van den Bergh JP , Szulc P , Cheung AM , Bouxsein M , Engelke K , Chapurlat R . The clinical application of high‐resolution peripheral computed tomography (HR‐pQCT) in adults: state of the art and future directions. Osteoporos Int. 2021;32(8):1465–1485. 34023944 10.1007/s00198-021-05999-z PMC8376700

[37] Putman MS , Yu EW , Lee H , et al. Differences in skeletal microarchitecture and strength in African‐American and white women. J Bone Miner Res. 2013;28(10):2177–2185. 23572415 10.1002/jbmr.1953 PMC3779478

[38] Wright NC , Looker AC , Saag KG , et al. The recent prevalence of osteoporosis and low bone mass in the United States based on bone mineral density at the femoral neck or lumbar spine. J Bone Miner Res. 2014;29(11):2520–2526. 24771492 10.1002/jbmr.2269 PMC4757905

[39] Looker AC , Borrud LG , Dawson‐Hughes B , Shepherd JA , Wright NC . Osteoporosis or low bone mass at the femur neck or lumbar spine in older adults: United States, 2005‐2008. NCHS Data Brief. 2012;93:1–8. 22617299

[40] Wright NC , Chen L , Saag KG , Brown CJ , Shikany JM , Curtis JR . Racial disparities exist in outcomes after major fragility fractures. J Am Geriatr Soc. 2020;68(8):1803–1810. 32337717 10.1111/jgs.16455 PMC7935465

[41] Finkelstein JS , Lee ML , Sowers M , et al. Ethnic variation in bone density in premenopausal and early perimenopausal women: effects of anthropometric and lifestyle factors. J Clin Endocrinol Metab. 2002;87(7):3057–3067. 12107201 10.1210/jcem.87.7.8654

[42] Looker AC , Sarafrazi Isfahani N , Fan B , Shepherd JA . Trabecular bone scores and lumbar spine bone mineral density of US adults: comparison of relationships with demographic and body size variables. Osteoporos Int. 2016;27(8):2467–2475. 26952009 10.1007/s00198-016-3550-6 PMC7593898

[43] Mui LW , Haramati LB , Alterman DD , Haramati N , Zelefsky MN , Hamerman D . Evaluation of vertebral fractures on lateral chest radiographs of inner‐city postmenopausal women. Calcif Tissue Int. 2003;73(6):550–554. 14517719 10.1007/s00223-003-0064-y

